# Expression of a new laccase from *Moniliophthora roreri* at high levels in *Pichia pastoris* and its potential application in micropollutant degradation

**DOI:** 10.1186/s13568-017-0368-3

**Published:** 2017-03-29

**Authors:** Agathe Bronikowski, Peter-Leon Hagedoorn, Katja Koschorreck, Vlada B. Urlacher

**Affiliations:** 1 Institute of Biochemistry II, Heinrich-Heine University Düsseldorf, Universitätsstraße 1, 40225 Düsseldorf, Germany; 2 Department of Biotechnology, Delft University of Technology, Julianalaan 67, 2628 BC Delft, The Netherlands; 3 Bioeconomy Science Center (BioSC), Heinrich-Heine University Düsseldorf, Universitätsstraße 1, 40225 Düsseldorf, Germany

**Keywords:** Laccase, Expression, *Pichia pastoris*, Micropollutant degradation

## Abstract

Laccases have gained significant attention due to their emerging applications including bioremediation, biomass degradation and biofuel cells. One of the prerequisites for the industrial application of laccases is their sufficient availability. However, expression levels of recombinantly expressed laccases are often low. In this study Mrl2, a new laccase from the basidiomycete *Moniliophthora roreri,* was cloned in *Pichia pastoris* and produced in an optimized fed-batch process at an exceptionally high yield of 1.05 g l^−1^. With a redox potential of 0.58 V, Mrl2 belongs to mid-redox potential laccases. However, Mrl2 demonstrated high k_cat_ values of 316, 20, 74, and 36 s^−1^ towards 2,2′-azino-bis(3-ethylbenzthiazoline-6-sulfonic acid) (ABTS), syringaldazine (SGZ), 2,6-dimethoxyphenol (2,6-DMP) and guaiacol, respectively. Mrl2 remained stable above pH 6 and in the presence of many metal ions, which is important for application in bioremediation. Mrl2 was investigated for the ability to degrade endocrine disrupting chemicals (EDCs) and non-steroidal anti-inflammatory drugs (NSDAIs) at neutral pH value. The enzyme accepted and converted estrone, 17β-estradiol, estriol, the synthetic contraceptive 17α-ethinyl estradiol and bisphenol A at pH 7 faster than high-potential laccases from *Trametes versicolor*. For example, within 30 min Mrl2 removed more than 90% bisphenol A, 17ß-estradiol, 17α-ethinyl estradiol and estriol, respectively. The concentration of the recalcitrant drug diclofenac dropped by 56% after 20 h incubation with Mrl2.

**Electronic supplementary material:**

The online version of this article (doi:10.1186/s13568-017-0368-3) contains supplementary material, which is available to authorized users.

## Introduction

Laccases (EC 1.10.3.2) are multi-copper oxidases which catalyze the oxidation of various electron-rich organic and inorganic molecules like mono- and diphenols, polyphenols, diamines, aminophenols, aromatic or aliphatic amines with the four-electron reduction of molecular oxygen to water (Thurston 1994; Xu 1996; Xu et al. 1996). Small compounds, so called redox mediators, can expand the substrate range to non-phenolic lignin derivatives (Bourbonnais and Paice 1990; Eggert et al. 1996) or recalcitrant dyes (Soares et al. 2001). Laccases contain four copper ions. Substrates are oxidized at the mononuclear type 1 (T1) copper site and electrons are transferred to the trinuclear site comprising one T2 and two T3 copper ions, where the reduction of oxygen to water takes place (Solomon et al. 2008). Laccases are ubiquitous and found in plants, fungi, insects (Mayer and Staples 2002), yeast (Kalyani et al. 2015) and bacteria (Dwivedi et al. 2011). The redox potential of laccases is quite different and range from 0.36 to 0.8 V. It is widely accepted that the axial ligand of the T1 copper as well as a tripeptide in the T1 copper site (LEA) roughly indicate the redox potential of laccases (Mot and Silaghi-Dumitrescu 2012; Xu et al. 1998). Low-potential laccases, mainly found in bacteria, with a redox potential from 0.36 to 0.46 V exhibit a methionine at the axial position, while middle-potential laccases, mainly from ascomycete origin, with a redox potential of 0.46–0.71 V usually have a leucine. High-potential laccases with a redox potential >0.71 V are mainly found in basidiomycetes. They display a phenylalanine as a non-coordinating axial ligand (Mot and Silaghi-Dumitrescu 2012). High-potential laccases typically possess a broader substrate spectrum than low- or middle-potential laccases and are even able to oxidize substrates with a redox potential of up to 1.2 or 1.4 V (Tadesse et al. 2008). Regarding their broad substrate spectrum while only relying on water as a cosubstrate, laccases often are called “green” biocatalysts (Pardo and Camarero 2015). Therefore, these enzymes, and high-potential laccases in particular, are attractive for industrial applications as in the pulp and paper, food and textile industry, nanobiotechnology, synthetic chemistry or bioremediation (Rodriguez Couto and Toca Herrera 2006).

Micropollutants comprising endocrine disrupting, toxic, persistent and bioaccumulative substances, like EDCs and NSAIDs (Table 1), are one of the key problems facing humanity (Schwarzenbach et al. 2006). They are ubiquitous in aquatic environments, since e.g. human or veterinary pharmaceuticals are only partially metabolized and eventually end up in the wastewater system (Hamid and Eskicioglu 2012). In an aging society it is very likely that more and more pharmaceuticals will be released in the environment. Human hormones as 17β-estradiol or the contraceptive 17α-ethinyl estradiol have been reported to lead to feminization or sexual disruption of fish (Chen et al. 2016a; Orn et al. 2016). Other estrogens as estrone which is predominant in menopausal women and estriol, a metabolite of estrone and 17β-estradiol, are also found in wastewaters (Auriol et al. 2007a, b). The diphenylmethane derivatives bisphenol A and bisphenol S used in plastics have endocrine mimicking properties (Ji et al. 2013; Vinas and Watson 2013). Diclofenac and naproxen are NSAIDs which show analgesic, antipyretic and anti-inflammatory effects. Because of more and more emerging concerns about the fate of EDCs and NSAIDs much effort has been made to develop alternative strategies to remove those micropollutants since conventional wastewater treatment processes do not meet the demands (Schroder et al. 2016). Among others laccases are capable of reducing estrogenic activity in wastewater (Suzuki et al. 2003; Tsutsumi et al. 2001). However, most high-redox potential laccases demonstrate low activity under neutral or basic pH conditions found in wastewater (Baldrian 2006). Another limitation for a broader laccase application is their rather low expression level in recombinant hosts.

**Table 1:** Chemical structures of endocrine disrupting chemicals and nonsteroidal anti-inflammatory drugs investigated in this study

Endocrine disrupting chemicals (EDCs)			
E1		E2	
EE2		E3	
BPA		BPS	
Nonsteroidal anti-inflammatory drugs (NSAIDs)			
Diclofenac		Naproxen	
	

*E1* estrone, *E2* 17ß-estradiol, *EE2* 17α-ethinyl estradiol, *E3* estriol, *BPA* bisphenol A, *BPS* bisphenol S

In this study, we identified and characterized a new laccase, Mrl2, from *Moniliophthora roreri*. Mrl2 was produced at exceptionally high levels in *Pichia pastoris* (1.05 g l^−1^) in a 3 l fed-batch fermentation process. High stability above pH 6 and resistance to many metal ions make this enzyme suitable for application in wastewater treatment. Amongst others, 17β-estradiol, 17α-ethinyl estradiol and diclofenac, which are listed by the European Union (EU) as dangerous compounds which should be monitored by the EU members (Schroder et al. 2016) could be degraded by Mrl2.

## Materials and methods

### Materials

Unless specified otherwise, all chemicals (of analytical grade or higher) and commercial proteins were acquired from AppliChem. (Darmstadt, Germany), Thermo Fisher Scientific Inc. (Waltham, Massachusetts, USA), Sigma-Aldrich (Schnelldorf, Germany), VWR (Darmstadt, Germany), Fermentas (St. Leon-Rot, Germany) or New England Biolabs (Ipswich, Massachusetts, USA).

### Cloning of *mrl2*

The gene for Mrl2 (NCBI Reference Sequence: XP_007855001.1) was codon optimized (GenBank accession number: KY111767) with JCat for expression in yeast (http://www.jcat.de/) and synthesized by Eurofins (Ebersberg Germany). The gene was cloned in the pPICZαA vector from Invitrogen™ (Carlsbad, California, USA) with both the native signal peptide and the α-factor secretion signal from *Saccharomyces cerevisiae*. Mrl2 was amplified with primers mrl2_BstBI_fw (GATA*ttcgaa*ATGGCTAGATTGCAATTC) and mrl2_XbaI_rev (CA*agatct*TTACAAGTCGTCGTCAG) and mrl2_*Xho*I_fw (GTAT*ctcaga* **AAAAGA**TCTATCGGTCCAATCG) and mrl2_*Xba*I_rev for the native signal sequence and the α-factor construct, respectively. Recognition sites for the endonucleases are underlined. The forward primer contained the Kex2 signal cleavage site (highlighted in bold), thus *mrl2* starts right behind Kex2 at the end of the alpha factor pre pro leader sequence. The vector and the amplified genes were subjected to cleavage with respective restriction enzymes and ligated into pPICZαA to generate pPICZαAmrl2 and pPICZAmrl2. The sequence of the constructs was confirmed by sequencing (GATC Biotech, Konstanz, Germany). pPICZαAmrl2 and pPICZAmrl2 were linearized with *Pme*I and inserted in *P.* *pastoris* X-33 (Invitrogen, Carlsbad, California, USA) by electroporation. After transformation, cells were selected at 30 °C on Yeast Extract Peptone Dextrose medium with sorbitol (YPDS; 10 g l^−1^ yeast extract; 182.2 g l^−1^ sorbitol; 20 g l^−1^ peptone; 15 g l^−1^ agar) agar plates with 100 µg ml^−1^ Zeocin™. For further assessment clones were streaked out on 2,2′-azino-bis(3-ethylbenzthiazoline-6-sulfonic acid) (ABTS)-Buffered Minimal Methanol (BMM; 13.4 g l^−1^ Yeast nitrogen base w/o amino acids; 15 g l^−1^ agar; 0.00004% biotin, 0.5% methanol; 100 mM potassium phosphate buffer pH 6; 0.3 mM CuSO_4_; 0.2 mM ABTS) agar plates and incubated at 30 °C. Clones which formed greenish halos were chosen for further experiments.

### Expression of Mrl2 in shaking flasks

*Pichia pastoris* transformants were grown in 10 ml Buffered Complex Glycerol medium (BMGY; 10 g l^−1^ yeast extract; 20 g l^−1^ peptone; 100 mM potassium phosphate buffer pH 6; 1% glycerol; 0.00004% biotin) over night at 30 °C and 180 rpm. Overnight cultures were used for inoculation of 50 ml BMM medium in baffled flasks to an OD_600_ of 0.1. The cultures were shaken at 30 °C and 180 rpm for 3 days. The medium was supplemented with methanol every day to 0.5% (v/v) final concentration. Samples were taken daily for cell density and laccase activity monitoring.

### Expression of Mrl2 in a 7.5 l bioreactor

*Pichia pastoris* transformants were grown in 10 ml BMGY (preculture) over night at 30 °C. A starter culture of 200 ml BMGY medium was inoculated with the preculture to an OD_600_ of 0.01–0.05 and grown over night at 30 °C and 180 rpm. The starter culture was used to inoculate a 7.5 l bioreactor (Infors, Bottmingen, Switzerland) containing 3 l fermentation basal salt medium (0.47 g l^−1^ CaSO_4_·2 H_2_O, 9.1 g l^−1^ K_2_SO_4_, 7.5 g l^−1^ MgSO_4_·7 H_2_O, 4.2 g l^−1^ KOH, 8 ml H_3_PO_4_ (85%), 50 g l^−1^, glycerol (87%), 0.87 mg l^−1^ biotin, 4.35 ml l^−1^ *Pichia* trace metals (PTM_1_, 6 g l^−1^ CuSO_4_·5 H_2_O, 0.08 g l^−1^ NaI, 3 g l^−1^ MnSO_4_·H_2_O, 0.5 g l^−1^ CoCl_2_, 20 g l^−1^ ZnCl_2_, 0.02 g l^−1^ H_3_BO_3_, 0.2 g l^−1^ Na_2_Mo_4_·2 H_2_O, 65 g l^−1^ FeSO_4_·7 H_2_O, 0.2 g l^−1^ biotin, 5 ml l^−1^ H_2_SO_4_) to an OD_600_ of 0.5. pH 6 was adjusted with 10% phosphoric acid and 25% ammonium hydroxide. In the first growth phase on glycerol the temperature was maintained at 30 °C. When glycerol was used up (noticeable in pO_2_ increase) expression was induced by addition of methanol (0.5% (w/v) containing 12 g l^−1^ trace metal salts PTM_1_) and temperature was shifted to 25 °C. Methanol was added automatically when the C-source was depleted, indicated by a sharp increase in pO_2_ value. 0.9 ml 1 M CuSO_4_ was added daily to the fermentation broth. Samples were taken daily for monitoring OD_600_, laccase activity and determining protein concentration by the Bradford assay using bovine serum albumin (BSA) as standard.

### Purification and characterization of Mrl2

The fermentation broth was centrifuged (10,000*g*; 15 min; 4 °C) and the supernatant was concentrated by Crossflow ultra-filtration with a cut-off membrane of 10 kDa (Pall, East Hills, NY, USA). The concentrated supernatant was centrifuged (22,000*g*; 20 min; 4 °C) and filtered through a 0.22 µm pore size filter. 5–10 ml of the concentrated supernatant was purified by DEAE FF anion exchange chromatography with an Äkta FPLC (GE Healthcare, Chalfont St Giles, Buckinghamshire, UK). After protein application the column was washed with 50 mM potassium phosphate buffer pH 6 and 200 mM NaCl. Mrl2 was eluted with 50 mM potassium phosphate buffer pH 6 and 250 mM NaCl. Blueish, active fractions (towards ABTS as substrate) were pooled and concentrated with a MILLIPORE Amicon^®^ stirred ultrafiltration cell 8200 (Bedford, Maine, USA) with a cut-off membrane of 10 kDa and desalted with a PD Midi Trap G-25 desalting column (GE Healthcare, Chalfont St Giles, Buckinghamshire, UK). Mrl2 was stored in 50 mM potassium phosphate buffer pH 6 at −20 °C until further use.

Protein concentration was estimated by the Bradford Assay with BSA as standard. Deglycosylation was conducted with PNGase F as described in the manual instruction. SDS-PAGE was conducted according to Laemmli (1970). Samples used for the zymogram were not heated at 95 °C. For activity staining the SDS-PAGE gel was incubated in 100 mM sodium acetate buffer pH 5 supplemented with 0.5 mM ABTS.

Copper content of Mrl2 was determined by atomic absorption spectroscopy on a Perkin Elmer AAnalyst 100 (Waltham, USA) equipped with an air-acetylene burner. Mrl2 was diluted in water for copper measurement. The same sample was used for protein concentration determination by Bradford assay and at 280 nm. For determining the protein concentration the molar extinction coefficient was calculated with http://web.expasy.org/protparam/as 80,580 M^−1^ cm^−1^.

Redox potential measurements were performed in a nitrogen flushed glove box under the absence of oxygen. For the titration redox mediator couples K_3_[Fe(CN)_6_]/K_4_[Fe(CN)_6_] and [Fe(bipy)_2_]^3+^/[Fe(bipy)_2_]^2+^ were used with a standard redox potential of 0.433 V (O’Reilly 1973) and 0.78 V versus the standard hydrogen electrode, respectively. For the bipyridine complex solutions, iron(III) chloride hexahydrate and iron(II) chloride were mixed in 1:2 ratio with 2,2′-bipyridine. The reduction status of the T1 copper was monitored at 600–750 nm. Measurements were performed in 50 mM potassium phosphate buffer, pH 7.5 at room temperature in duplicate. The normalized absorbance was plotted against the redox potential with the software Igor Pro (Wavemetrics; https://www.wavemetrics.com/) and the midpoint was determined using the Nernst equation for a one electron redox reaction: $Y = \frac{A}{{1 + e^{{\frac{nF}{RT}(E_{m} - E)}} }} + B$ with n = 1, F = 96,486 C mol^−1^, R = 8.314 J mol^−1^ K^−1^, T = 293 K, E_m_ = midpoint potential.

### Enzyme activity determination and kinetic parameters

Enzyme activity was determined in 100 mM sodium acetate buffer, pH 5 with 0.5 mM ABTS at room temperature. The increase of absorbance resulting from ABTS oxidation was monitored at 420 nm (ε = 36,000 M^−1^ cm^−1^). Kinetic parameters of Mrl2 with the substrates ABTS, 2,6-dimethoxyphenol (2,6-DMP; 468 nm; ε = 49,600 M^−1^ cm^−1^), guaiacol (470 nm; ε = 26,000 M^−1^ cm^−1^) and syringaldazine (SGZ; 530 nm; ε = 65,000 M^−1^ cm^−1^) were determined in 100 mM citrate phosphate buffer at their optimal pH. The substrate concentration ranges were 0.00781–1 mM, 0.000976–0.5 mM, 0.001–10 mM and 0.0001–10 mM for ABTS, SGZ, 2,6-DMP, and guaiacol, respectively. One Unit is defined as the amount of enzyme that converts 1 µmol substrate per minute.

### Effect of pH, temperature, metal ions and inhibitors on enzyme activity

For determining the optimal pH the assay was conducted at different pH values: pH 2 and 8–10 in 100 mM Britton Robinson buffer and pH 3–7 in 100 mM citrate phosphate buffer. For temperature stability investigations the enzyme was diluted to 500 µg ml^−1^ in 50 mM potassium phosphate buffer pH 7 in 0.2 ml reaction tubes. The tubes were incubated at 20, 30, 40, 50, 60, 70 and 80 °C in a PCR Cycler and the residual activity was measured with the standard ABTS activity assay. For pH stability study Mrl2 was diluted in 100 mM Britton Robinson buffer at pH 2-10 and incubated at room temperature. Aliquoted samples were used directly for measuring residual activity with the ABTS assay. The activity in the presence of metal ions as indicated in Table 4 was determined with ABTS as substrate at pH 3 in 100 mM citrate phosphate buffer. The enzyme was incubated with the metal ions for 5 min before the addition of ABTS. The metal ions were added as sulfates or in the case of calcium as nitrate. To assess the metal chelating effect of the citrate buffer used, enzyme activity was tested also in 100 mM HEPES buffer, pH 3 in the presence of 100 mM calcium, nickel, cobalt or zinc. Enzyme stability in the presence of 10 and 100 mM glycerin, acetonitrile, methanol, ethanol, 2-propanol, acetone, dimethyl sulfoxide (DMSO), and dimethylformamide (DMF) was determined as described for metal ion tolerance but in sodium acetate buffer pH 5. For determining activity in the presence of potential inhibitors the protein was incubated in 100 mM sodium acetate buffer pH 5 including 10–100 mM NaCl, 0.1–1% SDS or 0.1% sodium azide for 5 min at room temperature. After incubation time ABTS was added to the reaction mixture and oxidation was followed at 420 nm.

### EDC and NSDAI degradation

Stock solutions of 100 mM estrone, 17ß-estradiol, 17α-ethinyl estradiol, estriol, bisphenol A, bisphenol S, naproxen and diclofenac, respectively, were made in DMF. Degradation studies were carried out in 2 ml tubes at room temperature with a total reaction volume of 750 µl. The reaction solution contained 50 mM potassium phosphate buffer, pH 7 containing 10% ethanol (Sei et al. 2008), 100 µM EDCs or NSDAIs and 20 U ml^−1^ Mrl2. The same samples but containing heat-inactivated Mrl2 were used as a control. The reaction tubes were shaken in a rotating over-head incubator at room temperature. 100 µl samples were taken after 0, 0.5, 1, and 20 h and mixed with 3 µl 6 M HCl to stop the reaction. 100 µl Methanol was added, samples were centrifuged (12,300 *g*, 10 min) and analyzed by HPLC. Laccases from *Trametes versicolor* (Sigma Aldrich) were used for comparative degradation studies. The laccases were dissolved in 50 mM potassium phosphate buffer, pH 7, and protein concentration was determined with the Bradford assay using BSA as standard. The solution was stored at −20 °C until use. Degradation reactions with 30 µg ml^−1^ laccase (Mrl2 and from *T. versicolor*, respectively) were conducted in a total volume of 500 µl 50 mM potassium phosphate buffer, pH 7 containing 10% ethanol, 100 µM EDCs or NSDAIs. For HPLC analysis samples were prepared as described above. Samples were analyzed immediately.

### HPLC analysis

Samples were analyzed on a Shimadzu HPLC (Shimadzu, Duisburg, Germany) with a Chromolith^®^ C18 100 mm, 4.6 mm reversed phase column (Merck, Darmstadt, Germany). As mobile phase a mixture of 100% methanol (mobile phase A) and water supplemented with 0.1% formic acid (v/v) (mobile phase B) was used. 10–25 µl of sample were injected for analysis. Steroids were analyzed with an isocratic elution method. The methanol concentration was 55% for estrone, 60% for 17ß-estradiol and 17α-ethinyl estradiol and 33% for estriol. For the NSAIDs the following gradients were used: for bisphenol A in 10 min from 50% mobile phase A to 75% mobile phase A, from 10 to 12 min 100% mobile phase A, from 12 to 15 min 50% mobile phase A; for diclofenac in 10 min from 70% mobile phase A to 85% mobile phase A, from 10 to 12 min 100% mobile phase A, from 12 to 15 min 50% mobile phase A; for bisphenol A in 10 min from 25% mobile phase A to 75% mobile phase A, from 10 to 12 min 100% mobile phase A, from 12 to 15 min 50% mobile phase A and for naproxen in 10 min from 50% mobile phase A to 75% mobile phase A, from 10 to 12 min 100% mobile phase A, from 12 to 15 min 50% mobile phase A. The flow of the mobile phase was 1–2 ml min^−1^. The compounds were evaluated at 280 nm. Stock solutions of EDCs and NSAIDs dissolved in 50% methanol to a concentration of 100 mM were used as standards. Degradation of the chemical compounds was calculated as percentage of the initial (time point 0 h) peak area size.

## Results

### Identification of a putative high-potential laccase

The catalytic activity of laccases is influenced by the difference in the redox potential of a substrate and the type 1 copper (Xu 1996; Xu et al. 1996). Thus, in most cases, high-potential laccases accept a broader range of substrates than low-potential laccases (Tadesse et al. 2008). To identify a laccase with a high redox potential, a BLAST search was done with the motif HCHIDWHLEAGF, containing the coordinating histidines of the T1 and T3 copper ions, the LEA tripeptide and the non-coordinating axial ligand phenylalanine, that is highly conserved within high-potential laccases (Mot and Silaghi-Dumitrescu 2012). A gene with the NCBI Reference Sequence XP_007855001.1 was identified as a putative laccase from a fungus *Moniliophthora roreri* causing frosty pod rot in cacao. The new gene was designated *mrl2*.

### Cloning and recombinant expression of Mrl2 in shaking flasks

The gene *mrl2* was cloned with either its native signal sequence or with the α-mating factor signal sequence from *Saccharomyces cerevisiae* for secretion into the vector pPICZαA (Invitrogen, Carlsbad, California, USA) resulting in pPICZAmrl2 (nMrl2) and pPICZαAmrl2 (αMrl2), respectively. The constructs were used to transform *P. pastoris* X-33. Transformants were screened for laccase production on buffered minimal methanol agar plates containing ABTS. After overnight incubation at 30 °C transformants of both constructs formed strong green halos. Selected *Pichia* transformants were cultivated in buffered minimal methanol medium in shaking flasks at 30 °C. Activity of Mrl2 (towards ABTS) increased over 3 days of expression and reached 1850 U l^−1^ for nMrl2 and 2630 U l^−1^ for αMrl2. Since Mrl2, secreted via the α-factor, provided higher activity towards ABTS, this construct was used for laccase production in a 3 l fed-batch fermentation process.

### Fermentation and purification of Mrl2

Laccase production was evaluated in a 3 l high cell-density fed-batch fermentation process. The highest volumetric activity of 281,000 U l^−1^ was reached on day seven after induction with methanol. The fermentation broth was harvested on day eight after induction when a volumetric activity of about 272,800 U l^−1^ was measured. Cell-density reached OD_600_ = 460. Mrl2 was purified from concentrated supernatant by DEAE FF anion exchange chromatography to a specific activity of 248 U mg^−1^ towards ABTS which resulted in 1.33-fold purification. The yield of laccase calculated on the basis of the specific activity was 1.05 g l^−1^.

**Fig. 1:**
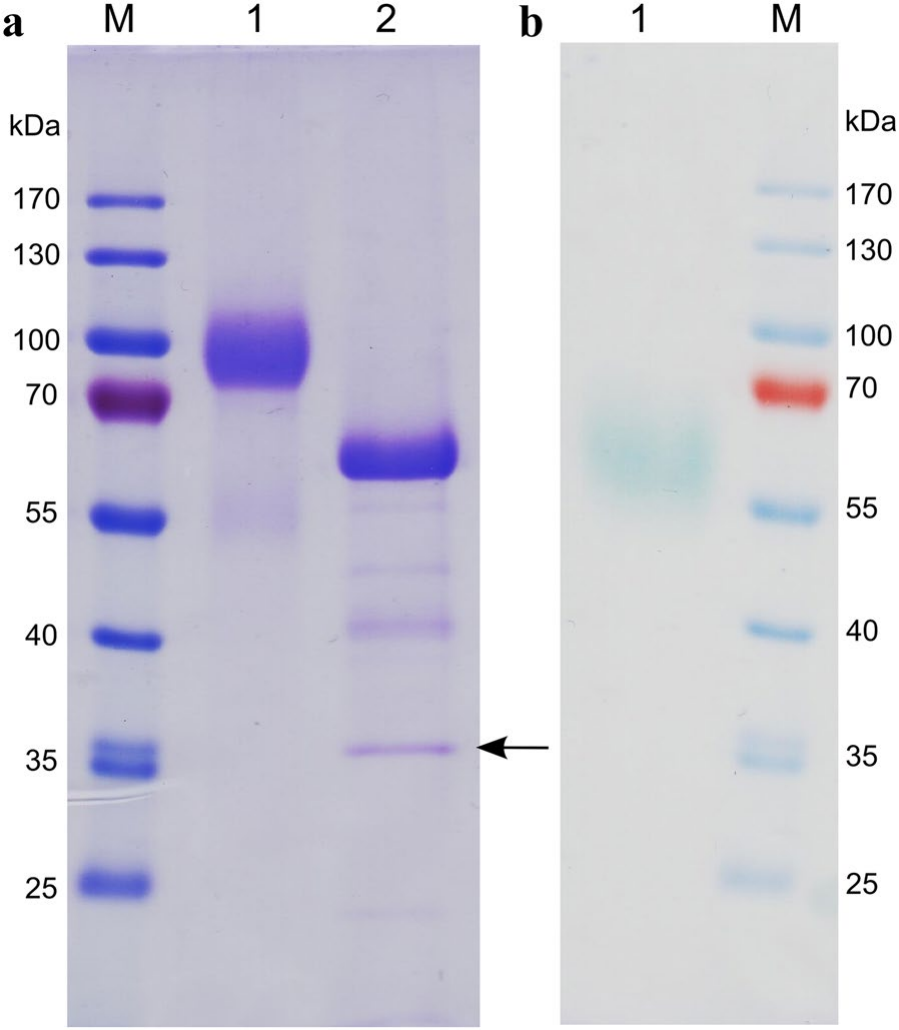
12.5% SDS-PAGE of Mrl2 purification. 5 µg total protein was loaded in each *lane*; *M* pre-stained protein markers; **a** Coomassie staining; *1* Mrl2 after DEAE FF purification, *2* Mrl2 after DEAE FF purification and PNGase F (indicated by an *arrow*) treatment, **b** zymogram with ABTS *1* purified Mrl2

SDS-PAGE of purified Mrl2 revealed a strong band around 90 kDa (Fig. 1). Deglycosylation with PNGase F resulted in a shift of the 90 kDa band to around 60 kDa. The calculated molecular weight of Mrl2 is 54 kDa. The discrepancy to the calculated molecular weight might be due to additional O-glycosylation of Mrl2, which is not cleaved off by PNGase F. Laccase zymogram in the gel supplemented with ABTS revealed a strong band at ~55 kDa (Fig. 1). Migration of Mrl2 in the zymogram at a molecular weight of ~55 kDa might result from faster migration of properly folded, unboiled Mrl2 sample loaded onto the gel. Boiled Mrl2 samples completely lost their activity in the zymogram.

### Biochemical characterization

**Fig. 2:**
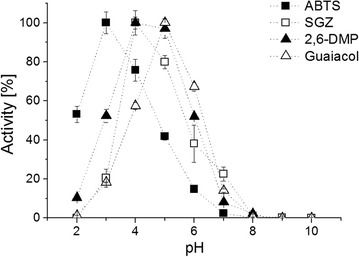
pH optimum of Mrl2 in reactions with the substrates ABTS, SGZ, 2,6-DMP and guaiacol. The highest measured activity for each substrate was set to 100%

Atomic absorption spectroscopy revealed fully copper loaded Mrl2 (4.8 copper ions per molecule). The pH optimum of Mrl2 was determined with four typical laccase substrates: ABTS, SGZ, 2,6-DMP and guaiacol. Mrl2 was rather active at acidic pH values (Fig. 2). For ABTS oxidation the optimal pH was 2, for SGZ and 2,6-DMP oxidation the pH optimum was 4 and the highest guaiacol oxidation was found at pH 5 (Fig. 2). Kinetic parameters were determined for all four substrates at their optimal pH value (Table 2). Mrl2-catalyzed reactions followed Michaelis–Menten kinetics. The highest k_cat_ value was determined with ABTS as substrate (316 s^−1^) and the lowest with SGZ (21 s^−1^). K_m_ values ranged from 12.45 µM for syringaldazine to 2235 µM for guaiacol.

**Table 2:** K_m_ and k_cat_ values of Mrl2 with ABTS, SGZ, 2,6-DMP and guaiacol at their corresponding pH optima

	K_m_ (µM)	k_cat_ (s^−1^)	k_cat_ K_m_^−1^ (µM^−1^ s^−1^)
ABTS	24.13 ± 1.9	316	13.076
SGZ	12.45 ± 1.6	21	1.684
2,6-DMP	358 ± 58.5	74	0.207
Guaiacol	2235 ± 117.5	37	0.016

**Fig. 3:**
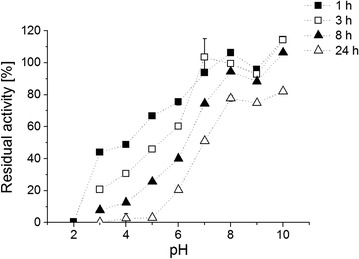
Stability of Mrl2 at different pH values. After 1 h (*black square*), 3 h (*white square*), 8 h (*black triangle*) and 24 h (*white triangle*) incubation the residual activity (indicated in % of initial activity) was determined

Stability of Mrl2 was determined at pH 2–10 and was found to be higher at basic pH values than at acidic ones. After 1 h incubation at pH 2 no activity of Mrl2 was detectable (Fig. 3). At pH 3–6 after 1 h incubation residual activity of 45–75% was found, while after 24 h the activity was hardly detectable. At pH 7 the activity was still around 50% after 24 h incubation and at pH 8–10 residual activity was highest with around 70% after 24 h (Fig. 3).

Half-life of Mrl2 at temperatures between 20 and 80 °C was determined at pH 7. Mrl2 was quite stable at 20 °C and pH 7 with a half-life of 13 days (Table 3). At elevated temperature the stability of Mrl2 was quite low with a half-life of 59 min at 50 °C and less than 1 min at 60 °C and above.

**Table 3:** Half-life of 500 µg ml^−1^ Mrl2 after incubation in 50 mM potassium phosphate buffer, pH 7 at different temperatures

Temperature (°C)	Half-life
20	13 days
30	3 days
40	12.5 h
50	59 min
60, 70, 80	<1 min

The effect of different metal ions, water-miscible organic solvents as well as some inhibitors on Mrl2 was examined by using ABTS as substrate. The metal ions were applied as sulfates, except Ca^2+^, which was applied as Ca(NO_3_)_2_ due to low solubility of CaSO_4_. Activity of Mrl2 was hardly or not affected by Mn^2+^, Co^2+^, Cu^2+^, Na^2+^, Zn^2+^ and K^+^ even at 100 mM concentration. At a concentration of 10 mM only Ni^2+^ and Ca^2+^ led to a slight inhibition of Mrl2 of 15–18%. At 100 mM metal ion concentration again Ni^2+^ and Ca^2+^ ions led to the highest inhibition of Mrl2 with residual activities towards ABTS of 26.2 and 50.4%, respectively. NO_3_^−^ was not responsible for the inhibitory effect of Ca(NO_3_)_2_ since 100 mM KNO_3_ had no effect on the activity of Mrl2 (data not shown). 100 mM Mg^2+^ ions diminished the activity by about 20%, while the other tested metal ions hardly reduced the activity but even increased it in the case of Mn^2+^ (Table 4).

**Table 4:** Residual activity of Mrl2 towards ABTS in presence of different metal ions, cosolvents and inhibitors

Metal ions	Residual activity (%)		Cosolvent	Residual activity (%)		Inhibitor	Concentration	Residual activity (%)
	10 mM	100 mM		10%	20%			
Mn^2+^	99.3 ± 1.9	119.4 ± 3.8	Ethanol	81.9 ± 1.5	21.7 ± 2.4	SDS	0.1%	100.3 ± 0.7
Co^2+^	100.9 ± 0.9	104.6 ± 2.9	Methanol	76.5 ± 2.7	39 ± 0.8		1%	77.7 ± 1.1
Ni^2+^	82.7 ± 1.5	26.2 ± 0.9	2-Propanol	61.9 ± 2.5	5.5 ± 2.5			
Ca^2+^	85.4 ± 1	50.4 ± 1.9	ACN	52 ± 2.8	0.7 ± 0.4	Sodium azide	0.1%	3.3 ± 1.9
Cu^2+^	104.9 ± 2.5	95.2 ± 2.7	DMSO	22.9 ± 2.2	26.1 ± 3.6			
Na^+^	99 ± 2.8	105.1 ± 4.4	Acetone	22.2 ± 2	16.6 ± 2.3	Cl^*−*^	10 mM	37.3 ± 0.9
Mg^2+^	101.5 ± 1.5	80.1 ± 1.7	DMF	40.2 ± 4.3	16.6 ± 0.4		50 mM	12.2 ± 0.15
Zn^2+^	104.9 ± 2.1	97.0 ± 1.2	Glycerol	85.7 ± 0.5	89.7 ± 1.9		100 mM	6.7 ± 0.04

*ACN* acetonitrile, *DMSO* dimethyl sulfoxide, *DMF* dimethylformamid

Water-miscible organic solvents more severely affected Mrl2 activity than metal ions. Already with 10% DMSO or acetone residual activity of Mrl2 dropped by about 80% (Table 4). 10% of ethanol and methanol slightly affected activity of Mrl2, while at a concentration of 20% residual activity of Mrl2 was 22 and 39%, respectively. 20% acetonitrile and 2-propanol inhibited Mrl2 almost completely. Activity of Mrl2 was hardly impaired by glycerol with about 90% residual activity at 20% glycerol.

Low concentrations of SDS had little effect on Mrl2 while 0.1% sodium azide almost completely inhibited Mrl2. Cl^−^ ions had a strong inhibitory effect on Mrl2. In the presence of 10 mM Cl^−^ Mrl2 showed 37% residual activity and at 50 and 100 mM concentration only 12.2 and 6.74% residual activity towards ABTS could be determined.

### Redox potential

For redox potential determination we used [Fe(CN)_6_]^3−^/[Fe(CN)_6_]^4–^and [Fe(bipy)_2_]^3+^/Fe[(bipy)_2_]^2+^ redox couples with a standard redox potential of 0.433 V (O’Reilly 1973) and 0.78 V, respectively. In first titrations with the [Fe(bipy)_2_]^3+^/Fe[(bipy)_2_]^2+^ redox mediator couple Mrl2 could not be fully reduced (data not shown), therefore the [Fe(CN)_6_]^3–^/[Fe(CN)_6_]^4–^ couple with the lower standard potential was applied. The redox titration with ferro- and ferricyanide could not fully oxidize Mrl2. For this reason the sample was aerated and the potential was set to an arbitrary high potential of 1.23 V. Redox titrations with iron(II/III) bipyridine indicated that the redox potential must be lower than 0.7 V. Titrations with the ferri/ferrocyanides confirmed these findings and revealed a redox potential of 0.58 V (Additional file 1: Figure S1).

### Degradation of EDCs and NSAIDs

**Fig. 4:**
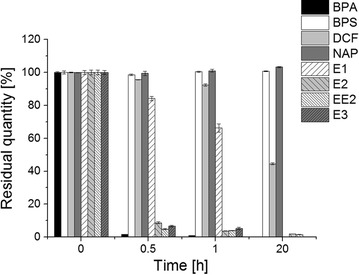
Residual quantity of EDCs and NSAIDs (%) after 0.5, 1 and 20 h of incubation with 20 U ml^−1^ Mrl2 at room temperature in 50 mM potassium phosphate buffer, pH 7. Measurements were carried out in triplicate

In the next step we examined the degradation of several micropollutants (Table 1) by Mrl2 at pH 7. Mrl2 degraded bisphenol A and the steroids 17ß-estradiol, 17α-ethinyl estradiol and estriol within 30 min below 10% residual concentration. Estrone seemed to be the most recalcitrant among the tested estrogens. After 1 h incubation a residual concentration of about 55% could be determined. After 20 h concentration of estrone decreased below the detection limit. Diclofenac was the most recalcitrant compound that could be degraded. After a reaction time of 20 h still about 42% diclofenac remained. Bisphenol S and naproxen were not affected by Mrl2 treatment within 20 h (Fig. 4).

**Fig. 5:**
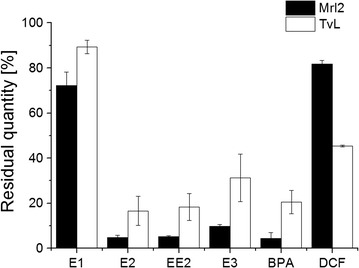
Residual quantity of EDCs and NSAIDs (%) after incubation with 30 µg ml^−1^ Mrl2 or TvL. The reaction time was set to 1 h except for DCF, for which it was 24 h. Measurements were carried out in triplicate

A comparative EDCs and NSAIDs degradation study was conducted with commercially available laccases preparation from *T. versicolor* (TvL). 30 µg ml^−1^ Mrl2 and TvL were used to degrade 100 µM micropollutants. After 1 h treatment, Mrl2 degraded all four estrogens and bisphenol A to a higher extent than TvL, while for diclofenac hardly any difference could be noticed between the two laccases. The reaction time for diclofenac was extended to 24 h. In this case TvL showed approximately twice the activity of Mrl2 (Fig. 5).

## Discussion

Laccases have gained significant attention due to emerging applications including biomass degradation, biofuel cells, biocatalysis and bioremediation (Cannatelli and Ragauskas 2016; Kudanga et al. 2011; Mikolasch and Schauer 2009; Senthivelan et al. 2016; Strong and Claus 2011; Suzuki et al. 2003). However, low expression levels have been recognized as a major obstacle to industrial use of these enzymes (Piscitelli et al. 2010). Our results demonstrate that Mrl2 was expressed at exceptionally high levels in *P. pastoris* in a 3 l fed-batch fermentation process. To our knowledge Mrl2 expression achieved the highest level of a fungal laccase in *P.* *pastoris* reported so far with 281,000 U l^−1^, which corresponds to 1.05 g Mrl2/l medium at a specific activity of 248 U mg^−1^. Similar laccase yield in *P. pastoris* was only reported for a bacterial laccase from *Thermus thermophilus* with 1.2 g l^−1^ (Liu et al. 2015a). Specific activity of this enzyme (1.12 U mg^−1^) was, however, much lower compared to Mrl2. Comparable laccase yields of 0.8–1 g l^−1^ of the heterologously expressed fungal laccase *T. versicolor* could only be reached using the filamentous fungal host *Trichoderma reesei* (Baker and White 2001). Higher yields were only reported for homologous expression of laccases in basidiomycetes (Table 5). Explanations for the exceptional high expression of Mrl2 in *P. pastoris* remain elusive. Both the α-factor and the native secretion signal sequence resulted in high secretion of Mrl2, indicating that high expression yield can rather be traced back to the laccase sequence than signal peptide used.

**Table 5:** Comparison of reported laccase yields and volumetric activity in different expression hosts

Source	Expression host	Laccase	Yield (mg l^−1^)	Vol. activity (U l^−1^)	Reference
Heterologous expression in yeast					
* Trametes versicolor*	*Pichia pastoris*			2.8	Bohlin et al. (2006)
* Cryphonectria parasitica*	*Saccharomyces cerevisiae*	lac3		41.5	Kim et al. (2010)
* Pleurotus eryngii*	*Saccharomyces cerevisiae*			146	Bleve et al. (2008)
* Melanocarpus albomyces*	*Saccharomyces cerevisiae*		3	168	Kiiskinen and Saloheimo (2004)
* Bacillus licheniformis*	*Pichia pastoris*			227.9	Lu et al. (2013)
* Moniliophthora perniciosa* FA553	*Pichia pastoris*	LacMP		232	Liu et al. (2015b)
* Melanocarpus albomyces*	*Saccharomyces cerevisiae*	MaL	7	270	Andberg et al. (2009)
* Ganoderma lucidum*	*Pichia pastoris*		580	685.8	You et al. (2014)
* Pycnoporus cinnabarinus*	*Yarrowia lipolytica*		19.84	1024	Madzak et al. (2005)
* Yarrowia lipolytica*	*Pichia pastoris*	YlLac		1290	Kalyani et al. (2015)
* Bacillus subtilis*	*Pichia pastoris*	CotA		1648.15	Wang et al. (2015)
* Ganoderma weberianum* TZC-1	*Pichia pastoris* GS115	GwLac1		2260	Zhou et al. (2014)
* Trametes trogii*	*Pichia pastoris*	Lcc1	17	2520	Colao et al. (2006)
* Botrytis aclada*	*Pichia pastoris*		517	3220	Kittl et al. (2012a)
* Ganoderma fornicatum* 814	*Pichia pastoris*	rLac1		3460	Huang et al. (2011)
* Lenzites gibbosa*	*Pichia pastoris*			5406	Zheng et al. (2014)
* Trametes* sp. AH 28-2	*Pichia pastoris*	lacA	4	5470	Hong et al. (2006)
* Rigidoporus microsporus* (*Fomes lignosus*)	*Pichia pastoris*		9.03	5950	Liu et al. (2003)
* Thermus thermophilus* SG0.5JP17-16	*Pichia pastoris*	LacTT	1200	6130	Liu et al. (2015a)
* Cerrena* sp. HYB07	*Pichia pastoris*	Lac1	19.3	6300	Yang et al. (2015)
* Pleurotus sajor*-*caju*	*Pichia pastoris*	lac4	110	10,200	Soden et al. (2002)
* Trametes versicolor*	*Pichia methanolica*			12,600	Guo et al. (2006)
* Trametes versicolor*	*Pichia pastoris*	lccA		18,123	Li et al. (2014)
* Botrytis aclada*	*Pichia pastoris*		495	51,000	Kittl et al. (2012b)
* Trametes versicolor*	*Pichia pastoris*			140,000	Hong et al. (2002)
* Trametes* sp. 420	*Pichia pastoris*		136	239,000	Zhou et al. (2007)
* Pycnoporus cinnabarinus*	*Pichia pastoris*		8		Otterbein et al. (2000)
* Myceliophthora thermophila*	*Saccharomyces cerevisiae*		18		Bulter et al. (2003)
Heterologous expression in filamentous fungi					
* Trametes versicolor*	*Aspergillus niger*			2700	Bohlin et al. (2006)
* Pycnoporus coccineus*	*Aspergillus oryzae*			3000	Hoshida et al. (2005)
* Trametes hirsute*	*Penicillium canescens*			3000	Abianova et al. (2010)
* Myceliophthora thermophila*	*Aspergillus oryzae*	r-MtL	19		Berka et al. (1997)
* Phlebia radiate*	*Trichoderma reesei*		20		Saloheimo and Nikupaavola (1991)
* Pycnoporus cinnabarinus*	*Aspergillus niger*		70		Record et al. (2002)
* Melanocarpus albomyces*	*Trichoderma reesei*		920		Kiiskinen et al. (2004)
* Trametes versicolor*	*Trichoderma reesei*		800–1000		Baker and White (2001)
Heterologous expression in bacteria					
* Bacillus pumilus* W3	*Bacillus subtilis* WB600	CotA		373,100	Guan et al. (2015)
Natural production host					
* Trametes versicolor* 1017	*Trametes versicolor* 1017	Tvlac		10,000	Chen et al. (2016b)
* Trametes* sp. AH 28-2	*Trametes* sp. AH 28-2	rLacB	31.6	32,000	Li et al. (2007)
* Trametes pubescens* MB 89	*Trametes pubescens* MB 89			65,000	Galhaup and Haltrich (2001)
* Trametes multicolor* MB 49	*Trametes multicolor* MB 49			85,000	Hess et al. (2002)
* Cerrena* sp. WR1	*Cerrena* sp. WR1	Lcc3	200	202,000	Chen et al. (2012)
* Cerrena* sp. HYB07	*Cerrena* sp. HYB07	LacA	108	210,800	Yang et al. (2014)
White rot fungus WR-1	White rot fungus WR-1			692,000	Revankar and Lele (2006)

Mrl2 possesses a phenylalanine as an axial ligand of the T1 copper. A positive correlation between hydrophobicity of this ligand and the redox potential E° has been reported (Marshall et al. 2009). Phenylalanine at this position implicates a high redox potential. Moreover, the hydrogen bond between serine at position 113 and glutamate at position 455 contributes to a long T1 Cu-His ligand distance which, according to Piontek and colleagues, strongly anticipates a high redox potential (Piontek et al. 2002). With a redox potential of 0.58 V, Mrl2, however, belongs to the middle-potential laccases. Besides the nature of the axial ligand and neighboring amino acids, redox potential is obviously influenced by other factors including hydrogen bonding of H(N)backbone and the coordinating SCys as well as protein and solvent dipoles and solvent accessibility (Hong et al. 2011). The reason why Mrl2 has a middle redox potential is under further investigation.

Important prerequisites for application of laccases in bioremediation for example in wastewater treatment are besides their sufficient availability, high stability at pH 7 or higher and the ability of degrading micropollutants including pharmaceuticals under neutral or slightly alkaline conditions even without adding redox mediators. In respect of these factors, Mrl2 looks very promising. Moreover, Mrl2 demonstrated high stability in the presence of metal ions which are often present in wastewater. Even at 100 mM concentration Mn^2+^, Co^2+^, Cu^2+^, Na^2+^, Zn^2+^ and K^+^ had almost no effect on laccase activity, examined in citrate phosphate buffer pH 3. A chelating effect of citrate on metal ions and thus their weaker effect on the laccase activity could be excluded since measurements in HEPES buffer provided comparable results (data not shown).

Despite middle redox potential, Mrl2 was able to degrade bisphenol A to 100% and all tested estrogens to more than 97% at neutral pH, with estrone being the most recalcitrant one. Diclofenac that is considered as poorly degradable in studies dealing with its removal (Barbosa et al. 2016), was degraded to 56%. Several reports describe effective removal of EDCs and NSAIDs by other laccases as well. Those experiments were carried out at acidic pH values, due to higher laccase activities at these pH values (Asadgol et al. 2014; Garcia-Morales et al. 2015; Macellaro et al. 2014; Sei et al. 2008; Tsutsumi et al. 2001). However, wastewater of plant effluents, which are considered the main source of estrogens (Snyder et al. 2001), usually show a neutral or basic pH. For this reason we used pH 7 for removal experiments with Mrl2. Despite the fact that fungal laccases usually demonstrate very low activities at that pH, Mrl2 still showed reasonable activity, thus representing an effective biocatalyst for the use in bioremediation. For example, Saito et al. (2004) used 50,000 U l^−1^ purified laccase from an ascomycete fungus belonging to *Chaetomiaceae* family to degrade 93.7% bisphenol A within 1 h, whereas 20,000 U l^−1^ Mrl2 degraded 98% bisphenol A within 30 min. To degrade the recalcitrant NSAID diclofenac Lloret et al. (2010, 2013) used 2000 U l^−1^ mid-redox potential laccase MtL from *Myceliophthora thermophila* and 2000 U l^−1^ (147 mg l^−1^) of high-redox potential *T. versicolor*. After 8 h incubation with MtL and 24 h incubation with *T. versicolor* laccase 2 and 27% diclofenac was removed, respectively, while 20,000 U l^−1^ (80 mg l^−1^) Mrl2 decreased diclofenac concentration by 8 and 56% after 1 and 20 h incubation, respectively. Lloret et al. (2010) also tested 2000 U l^−1^ MtL with the estrogens estrone, 17ß-estradiol and 17α-ethinyl estradiol at pH 7. While removal of 17ß-estradiol with Mrl2 and MtL was comparable (96 and 99% decrease for Mrl2 and MtL after 1 and 8 h, respectively), the contraceptive 17α-ethinyl estradiol was degraded by Mrl2 (95%, 1 h) much faster than by MtL (85%, 8 h).

Yet comparison of different micropollutant removal experiments might be misleading in regard to different specific activities of the laccases and thereby quite different amounts of applied enzyme as e.g. Mrl2 has an exceptionally high specific activity towards ABTS. This is why we applied commercial TvL and Mrl2 in a degradation experiment of several micropollutants at equal enzyme amounts. Though TvL possess a high redox potential (Reinhammar 1972), Mrl2 showed faster degradation of estrogens and bisphenol A within 1 h. Besides laccases’ redox potential, amino acids involved in substrate binding near the T1 copper site contribute to high substrate turnover rates. Our findings suggest better binding of hydroxylated compounds like estrogens and bisphenol A by Mrl2 than TvL. With the more recalcitrant, non-hydroxylated diclofenac, TvL displayed about 2.5-fold higher activity than Mrl2. In this case the higher redox potential of TvL might afford faster degradation of diclofenac compared to Mrl2. Another aspect might be different stability of the enzymes during 24 h incubation.

In concordance with previous studies, bisphenol S and naproxen were not degraded by laccase alone (Lloret et al. 2010). Laccase-mediated removal of bisphenol S and naproxen seems to require addition of small redox mediators, applied to expand the substrate spectrum of laccases to e.g. compounds with higher redox potentials like non-phenolic substances. These mediators are, however, mostly expensive and often not environmentally friendly, and their application in wastewater treatment thus doubtful.

In conclusion, Mrl2, a new laccase from *M. roreri* was identified and showed exceptionally high expression levels during fed-batch fermentation of recombinant *P. pastoris* (280,000 U l^−1^; 1.05 mg l^−1^) matching those of filamentous fungi. The enzyme is stable up to 30 °C, at alkaline pH values and active in the presence of several water-miscible organic solvents and metal ions. Despite its redox potential of 0.58 V Mrl2 degrades estrogens like estrone, 17β-estradiol, estriol and the contraceptive 17α-ethinyl estradiol and an estrogenic active substance bisphenol A faster than TvL at neutral pH. This makes Mrl2 a promising candidate for application in wastewater treatment.

## Additional file

**Additional file 1: Figure S1.** Additional information.

## Abbreviations

SDS-PAGE: sodium dodecyl sulfate–polyacrylamide gel electrophoresis
kDa: kilo Dalton
PNGase F: peptide-*N*-Glycosidase F
HPLC: high performance liquid chromatography
BMGY: buffered complex glycerol medium
BMM: buffered minimal methanol
DMSO: dimethyl sulfoxide
DMF: dimethylformamide
TvL: laccases preparation from *Trametes versicolor*
PTM_1_: *Pichia* trace metal salts
ABTS: 2,2′-azino-bis(3-ethylbenzthiazoline-6-sulfonic acid)
SGZ: syringaldazine
2,6-DMP: 2,6-dimethoxyphenol
HEPES: 2-[4-(2-hydroxyethyl)piperazin-1-yl]ethanesulfonic acid
DEAE FF: diethylaminoethyl-Sepharose Fast Flow
BPA: bisphenol A
BPS: bisphenol S
DCF: diclofenac
NAP: naproxen
E1: estrone
E2: 17ß-estradiol
EE2: 17α-ethinyl estradiol
E3: estriol
EDC: endocrine disrupting compound
NSAID: non-steroidal anti-inflammatory drug
