# Supplementary material for: Expression of a new laccase from Moniliophthora roreri at high levels in Pichia pastoris and its potential application in micropollutant degradation
Source: AMB Express. 2017 Mar 29;7:73. doi: 10.1186/s13568-017-0368-3 (PMC5371579; doi:10.1186/s13568-017-0368-3)
Supplement: Supplementary file 1 — Additional file 1: Figure S1. Additional information. [file 13568_2017_368_MOESM1_ESM.pdf]

Expression of a new laccase from *Moniliophthora roreri* at high levels in *Pichia pastoris* and its potential application in micropollutant degradation

Agathe Bronikowski[1], Peter-Leon Hagedoorn[2], Katja Koschorreck[1], Vlada B. Urlacher[1,*]

[1]Institute of Biochemistry and Bioeconomy Science Center (BioSC), Heinrich-Heine University Düsseldorf, Universitätsstraße 1, 40225 Düsseldorf, Germany

[2]Department of Biotechnology, Delft University of Technology, Julianalaan 67, 2628 BC Delft, The Netherlands

* Corresponding author
Vlada B. Urlacher
Institute of Biochemistry II
Heinrich-Heine University Düsseldorf
Universitätsstraße 1
40225 Düsseldorf, Germany
Tel.: +49-211-8113889
Fax.: +49-211-8113117

e-mail: vlada.urlacher@uni-duesseldorf.de

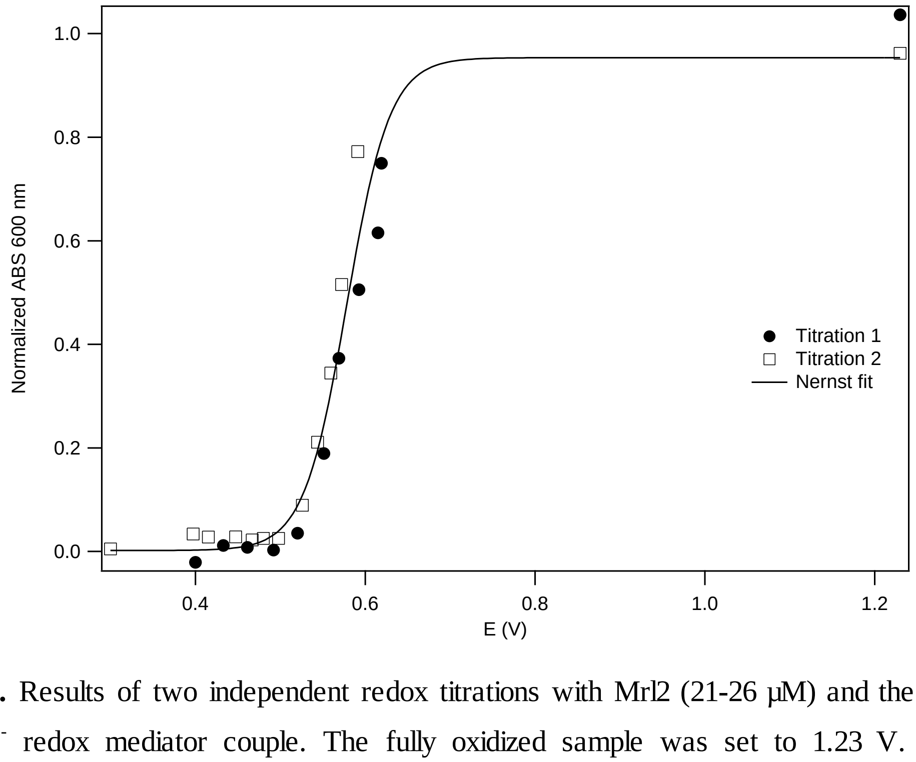

Figure S1. Results of two independent redox titrations with Mrl2 (21-26 µM) and the $[Fe(CN)_6]^{3-}$ / $[Fe(CN)_6]^{4-}$ redox mediator couple. The fully oxidized sample was set to 1.23 V. Fit to Nernst curve: $Y = \frac{A}{1+e^{\frac{nF}{RT}(E_m - E)}} + B$. Fixed parameters: n = 1; T = 293 K. Fit parameters: A = 0.95 ± 0.04; B = 0.00 ± 0.02; E_m = 0.578 ± 0.004 V.
